# Diagnostic Ability of Retinal Nerve Fiber Layer Thickness Deviation Map for Localized and Diffuse Retinal Nerve Fiber Layer Defects

**DOI:** 10.1155/2017/8365090

**Published:** 2017-01-10

**Authors:** Joong Won Shin, Mincheol Seong, Jung Wook Lee, Eun Hee Hong, Ki Bang Uhm

**Affiliations:** ^1^Department of Ophthalmology, Hanyang University College of Medicine, Seoul, Republic of Korea; ^2^Department of Ophthalmology, College of Medicine, University of Ulsan, Asan Medical Center, Seoul, Republic of Korea; ^3^Department of Ophthalmology, Hanyang University Guri Hospital, Hanyang University College of Medicine, Guri, Republic of Korea

## Abstract

*Purpose*. To evaluate the diagnostic ability of the retinal nerve fiber layer (RNFL) deviation map for glaucoma with localized or diffuse RNFL defects.* Methods*. Eyes of 139 glaucoma patients and 165 healthy subjects were enrolled. All participants were imaged with Cirrus HD-OCT (Carl Zeiss Meditec, Dublin, CA, USA). A RNFL defect was defined as at least 10 contiguous red (<1% level) superpixels in RNFL deviation map. The area, location, and angular width of RNFL defects were automatically measured. We compared sensitivities, specificities, and area under the receiver operating characteristic curves (AUCs) of RNFL deviation map and circumpapillary RNFL thickness for localized and diffuse RNFL defects. Subgroup analysis was performed according to the severity of glaucoma.* Results*. For localized defects, the area of RNFL defects (AUC, 0.991; sensitivity, 97%; specificity, 90%) in deviation map showed a higher diagnostic performance (*p* = 0.002) than the best circumpapillary RNFL parameter (inferior RNFL thickness; AUC, 0.914; sensitivity, 79%; specificity, 92%). For diffuse defects, there was no significant difference between the RNFL deviation map and circumpapillary RNFL parameters. In mild glaucoma with localized defect, RNFL deviation map showed a better diagnostic performance than circumpapillary RNFL measurement.* Conclusions*. RNFL deviation map is a useful tool for evaluating glaucoma regardless of localized or diffuse defect type and has advantages over circumpapillary RNFL measurement for detecting localized RNFL defects.

## 1. Introduction

Glaucoma is an optic neuropathy characterized by progressive injury to the retinal ganglion cells that results in loss of the retinal nerve fiber layer (RNFL). Evaluation of the RNFL is one of the most important clinical examinations for diagnosing glaucoma. Red-free RNFL photography has been a useful tool for detecting RNFL defects [1]. However, qualitative or semiquantitative assessment of RNFL using photography is dependent on the examiner's experience.

Glaucomatous RNFL loss can present as a localized wedge-shaped defect or a diffuse defect. Compared with a localized RNFL defect, diffuse RNFL atrophy is more difficult to detect and its diagnosis requires an experienced observer [1, 2]. Moreover, it is hard to clearly define the border of diffuse atrophy. This is somewhat unfortunate because diffuse RNFL atrophy is thought to be more common than localized defect, and presents in approximately 50% of cases compared with 30% for localized defects [1, 2].

Recent advances in ocular imaging technologies such as optical coherence tomography (OCT) allow objective and quantitative evaluation of the RNFL [3]. Topographic RNFL thickness mapping has become possible using high-speed and high-resolution imaging of spectral-domain OCT [4]. Abnormal RNFL measurements less than the lower 95th or 99th percentile at the 6 × 6 mm^2^ parapapillary region are displayed in the RNFL deviation map. The RNFL deviation map can visualize the distribution pattern of RNFL defects similar to that observed in fundus photographs [5] and there is a high topographic correlation of RNFL defects between red-free photography and the RNFL deviation map [6]. Most studies have investigated the diagnostic performance of OCT in glaucoma patients with localized RNFL loss identified on red-free RNFL photography [7, 8].

In this study, we identified localized and diffuse RNFL defects quantitatively and objectively using the RNFL deviation map of spectral-domain OCT. We evaluated and compared the diagnostic ability of RNFL deviation map and circumpapillary RNFL measurement for glaucoma with localized or diffuse RNFL defects.

## 2. Materials and Methods

### 2.1. Participants

A total of 139 glaucoma patients and 165 normal control subjects who visited either the general healthcare clinic or the glaucoma clinic at Hanyang University Medical Center from September 2012 to January 2013 were enrolled. All subjects underwent a comprehensive ophthalmic examination, which included a visual acuity test, slit-lamp biomicroscopy, Goldmann applanation tonometry, gonioscopy, refraction, fundus examination, pachymetry (SP-3000; Tomey, Nagoya, Japan), standard automated perimetry (Humphrey Field analyzer with SITA standard 30-2 test; Carl Zeiss Meditec, Dublin, CA, USA), red-free fundus photography (TRC-50IX; Topcon, Tokyo, Japan), and RNFL imaging with spectral-domain OCT (Cirrus HD-OCT; Carl Zeiss Meditec).

Normal and glaucoma subjects were included if they had a best-corrected visual acuity of ≥20/30, a spherical refractive error within the range of −6.0 to +3.0 diopters (D), and a cylinder correction within +3 D. Subjects with any ophthalmic or neurologic disease known to affect RNFL thickness or visual function were excluded. Normal subjects had a normal anterior segment on slit-lamp examination, normal visual field, normal appearing optic disc head, no RNFL defects, and no history of intraocular pressure > 21 mmHg. Glaucoma subjects had RNFL defects on red-free photographs or glaucomatous appearance of the optic nerve head on color fundus photographs (neuroretinal rim notching or thinning, or optic disc hemorrhage) and visual field defects that corresponded to the RNFL defects or optic nerve head abnormalities. Glaucoma subjects were selected from the patients who had stable visual field during long-term follow-up. Visual field defects were defined as (1) a cluster of ≥3 nonedge contiguous points with probabilities of <5% on the pattern deviation plot, at least one of which was depressed below the 1% level, (2) glaucoma hemifield test results outside normal limits, or (3) a pattern standard deviation (PSD) with a *p* value < 5% as confirmed by at least two reliable examinations. The severity of glaucomatous damage was classified into mild (mean deviation ≥ −6 dB) and moderate-to-severe (mean deviation < −6 dB). The visual field tests were considered to be reliable based on fixation losses and false-positive and false-negative results of 15% or less.

The study protocol was approved by the institutional review board of Hanyang University Medical Center and adhered to the tenets of the Declaration of Helsinki. Informed consent was obtained from all participants before participation.

### 2.2. Retinal Nerve Fiber Layer Imaging with Spectral-Domain Optical Coherence Tomography

Spectral-domain OCT imaging was performed with the Cirrus HD-OCT (software version 5.1). An Optic Disc Cube scan measured the RNFL thickness of 200 × 200 axial scans (pixels) in the 6 × 6 mm^2^ optic disc region. Abnormal RNFL measurements less than the lower 95th percentile range at the 6 × 6 mm^2^ parapapillary area were displayed in the RNFL thickness deviation map. The RNFL thickness deviation map is composed of 50 × 50 superpixels (each superpixel is composed of 4 × 4 pixels). Each superpixel was coded in yellow or red if the RNFL measurement was less than the lower 95th or 99th percentile range, respectively. The average, superior, nasal, inferior, temporal quadrant, and 12 clock-hour RNFL thicknesses were measured at a 3.46 mm diameter scan circle. Each sector was coded in green, yellow, or red for RNFL measurement greater than the lower 95th percentile, less than the lower 95th percentile, or less than the lower 99th percentile range, respectively. All images had signal strength of at least 7. Images with motion artifacts were rescanned at the same visit. RNFL segmentation was checked for every OCT image. The optic disc margin from OCT built-in algorithm was compared with clinical optic disc margin from optic disc photograph. Obvious discrepancy between two disc margins was excluded.

### 2.3. Quantification of Retinal Nerve Fiber Layer Defects

In this study, a RNFL defect was defined as at least 10 contiguous red (<1% level) superpixels in the RNFL deviation map [5] because the yellow (<5% level) coded area tends to overestimate RNFL defects or contains false-positive errors [6, 9]. The area, location, and angular width of RNFL defects were measured by a computer program written using MATLAB software (The MathWorks, Inc., Natick, MA, USA). Clusters of ≥10 contiguous red superpixels in the RNFL deviation map were automatically detected and outlined by a computer program according to the RGB (red, green, and blue) information. The center of the outlined object was calculated by the mathematical equation of the center of mass:

(1) R=1M∑i=1nmiri=1Area of RNFL defect·∑i=1nArea of each pixeli·Coordinates of each pixeli,

where *R* is center of mass, *M* is sum of the masses, *m*_*i*_ is mass of particle, and *r*_*i*_ are coordinates of particle.

**Figure 1:**
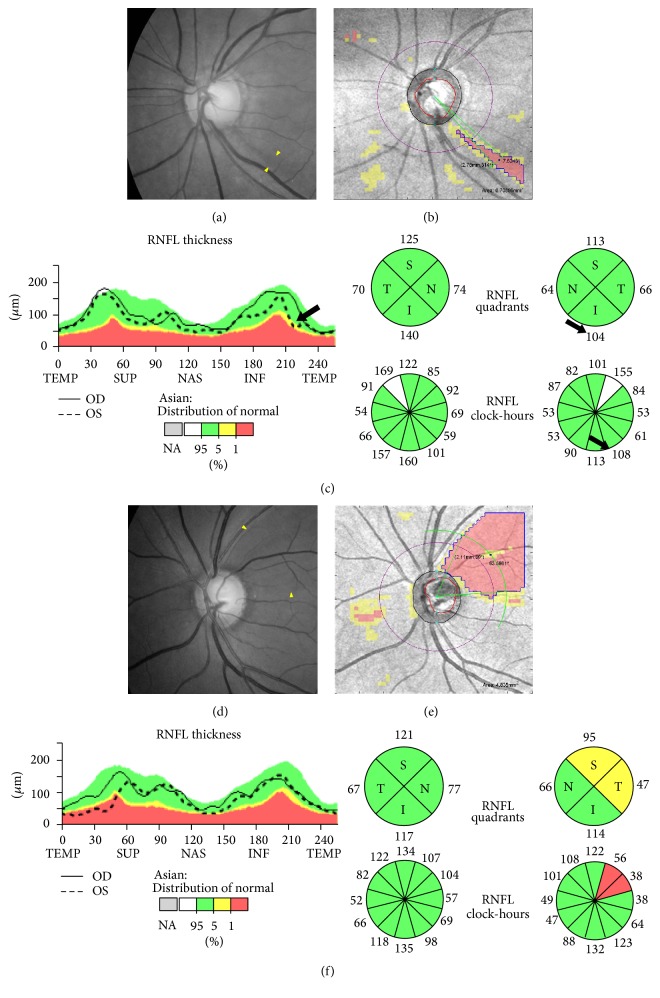
(a) Localized wedge-shaped RNFL defect (yellow arrowheads) was observed at the inferotemporal region in red-free photography. (b) RNFL defect (blue line) was defined as at least 10 contiguous red (<1% level) superpixels in the RNFL deviation map. The center (black dot) of the RNFL defect was calculated with polar coordinates [*r* = 2.78 mm, *θ* = 314°] (*r*: distance from the center of the optic disc, *θ*: angle from a temporal equator in a clockwise direction in right eyes and in a counterclockwise direction in left eyes). The angular width of the RNFL defect was determined as 7.52° where the boundary (blue line) of the RNFL defect met the circle (green arc) passing through the center of the RNFL defect. This was classified as a localized RNFL defect because of the angular width <30°. (c) In circumpapillary RNFL measurements, quadrant and clock-hour maps could not detect abnormalities at the corresponding area, although the TSNIT graph yielded suspicious downslope (black allows). (d) Diffuse RNFL defect (yellow arrowheads) was observed at the superotemporal region in red-free photography. The lower border was quite unclear for determination of the boundary. (e) The lesion was located at [2.11 mm, 39°] and corresponded to diffuse RNFL defect with angular width of 63.58°. (f) For a diffuse RNFL defect, circumpapillary RNFL measurements detected abnormalities at the corresponding area relatively well.

The center of RNFL defect can represent the location of the RNFL defect. The center of the RNFL defect was described with polar coordinates [*r*, *θ*], in which each point on a plane is determined by a distance (*r*) from the center of the optic disc and an angle (*θ*) from a temporal equator. The built-in algorithm of Cirrus HD-OCT provided the coordinate of the optic disc center in the printout results (e.g., “Disc Center [−0.02, 0.04] mm”). Temporal equator was defined as 9 o'clock from the optic disc center in right eyes and 3 o'clock in left eyes. Angles were measured in a clockwise direction in right eyes and in a counterclockwise direction in left eyes, with the temporal equator set at 0°. The angular width of RNFL defects was determined where the boundary of the RNFL defect met the circle passing through the center of the RNFL defect. A diffuse RNFL defect was defined as having an angular width > 30° [5]. To avoid false-positive errors, we excluded the red-coded area with center angle location 135° < *θ* < 225° (nasal quadrant), even if it exceeded 10 contiguous superpixels. These processes were described in Figure 1.

Multiple RNFL defects were dealt with according to the following criteria. (1) If multiple RNFL defects were detected in superior or inferior hemifield, localized or diffuse RNFL defects were determined by the sum of the angular width within each hemifield. (2) If RNFL defects were found as localized type in one hemifield and diffuse type in opposite side, we selectively analyzed the diffuse RNFL defect. (3) If RNFL defects were found as same type (“localized + localized” or “diffuse + diffuse”) in both superior and inferior hemifield, we randomly chose one of them.

### 2.4. Statistical Analyses

Subject demographics and quantitative measurements of RNFL defect were compared between the normal and glaucoma groups or between localized and diffuse RNFL defect groups using an independent* t*-test and Pearson chi-squared test. Area under the receiver operating characteristic curve (AUC) was used to describe the ability of circumpapillary RNFL thickness and area of RNFL defect to differentiate between normal eyes and glaucomatous eyes with localized or diffuse RNFL defects. Significant differences between AUCs were assessed using the method described by DeLong et al. [10]. *p* values of 0.05 or less were considered statistically significant. Statistical analyses were performed using MedCalc software (Version 12.2.1, Mariakerke, Belgium).

## 3. Results

Table 1 presents subject demographics. There were significant differences in mean deviation, pattern standard deviation, and rim area between the normal and glaucoma groups. Among 139 glaucoma patients, 62 (44.6%) had localized RNFL defects, 71 (51.1%) had diffuse RNFL defects, and 6 (4.3%) had no defect in the RNFL deviation map. Diffuse RNFL defects showed more severe visual field defects and neuroretinal rim thinning than localized defects (*p* < 0.001). The ratio of mild : moderate to advanced visual field defects was 49 : 13 in the localized defect group and 36 : 35 in the diffuse defect group (*p* < 0.001).

AUC values, sensitivity, and specificity of circumpapillary RNFL thickness and area of RNFL defect are presented in Table 2. The best discriminants of circumpapillary RNFL measurements were inferior RNFL thickness (AUC, 0.914; sensitivity, 79%; specificity, 92%) in the localized RNFL defect group and average RNFL thickness (AUC, 0.986; sensitivity, 97%; specificity, 96%) in the diffuse RNFL defect group. There was a significant difference between the best circumpapillary parameter of localized defects and that of diffuse RNFL defects (*p* = 0.010). The area of localized RNFL defects (AUC, 0.991; sensitivity, 97%; specificity, 90%) showed a higher diagnostic performance than the best circumpapillary parameter of localized defects (*p* = 0.002), but the area of diffuse RNFL defects (AUC, 1.000; sensitivity, 100%; specificity, 100%) showed no significant difference compared to the best circumpapillary parameter of diffuse defects (*p* = 0.249).

In mild glaucoma, a similar tendency of diagnostic performance for localized and diffuse RNFL defects was observed. The area of localized RNFL defects (AUC, 0.989; sensitivity, 96%; specificity, 90%) showed better diagnostic performance (*p* = 0.003) than inferior RNFL thickness (AUC, 0.896; sensitivity, 78%; specificity, 93%). However, the area of diffuse RNFL defects (AUC, 1.000; sensitivity, 100%; specificity, 100%) showed no significant difference (*p* = 0.252) from average RNFL thickness (AUC, 0.973; sensitivity, 94%; specificity, 96%). In moderate-to-severe glaucoma, there were no significant differences of diagnostic performance between RNFL deviation map (AUCs of red-coded area, 0.998 and 1.000 in localized and diffuse defect) and circumpapillary RNFL thickness (AUCs of best parameters, 0.983 and 1.000 in localized and diffuse defect).

**Figure 2:**
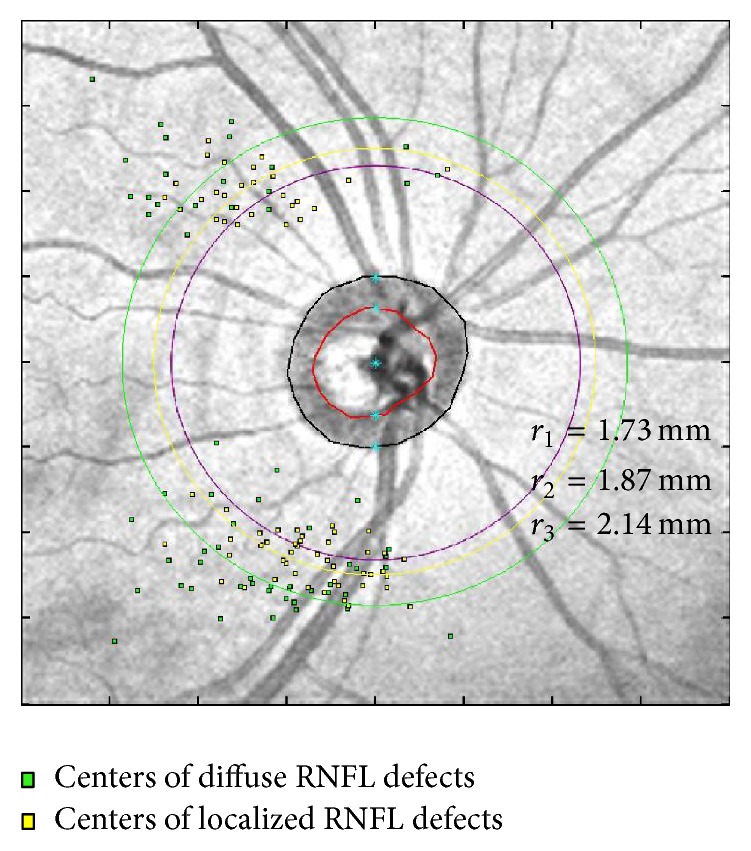
The distribution of centers of localized (green dots) and diffuse (yellow dots) RNFL defects. The average location of diffuse RNFL defects (*r*_2_; yellow circle) was significantly closer to the center of the optic disc than that of localized RNFL defect (*r*_3_; green circle). The purple circle was the original scanning position with radius of 1.73 mm.

The distribution and location of the centers of RNFL defects are presented in Figure 2 and Table 3. Average distance between the center of the RNFL defect and the center of the optic disc was 2.14 ± 0.42 mm in the localized defect group and 1.87 ± 0.24 mm in the diffuse defect group. Diffuse RNFL defects were significantly closer to the center of the optic disc than localized RNFL defects (*p* < 0.001). The average distance was outside the original circle scan line (radius = 1.73 mm) of conventional RNFL measurements. There was no significant difference in angular location between the two groups.

## 4. Discussion

Diffuse RNFL defects have been difficult to quantify by red-free photography, because recognition of the change in RNFL reflectivity and striation pattern is too vague or subjective to allow clear identification of the border of RNFL atrophy. A few studies have attempted to evaluate diffuse RNFL defects in red-free photography but had limitations in presenting the topographic boundary information [11–13]. The RNFL deviation map offers an effective approach to quantify multiple dimensions of RNFL defects (thickness, area, angular location, and angular width) [9]. We objectively identified the boundary of RNFL defects using the RNFL deviation map and classified the defects as localized and diffuse type. The diagnostic ability for localized and diffuse RNFL defects was evaluated in circumpapillary RNFL measurement and RNFL deviation map.

In this study, we found a significant difference in the diagnostic ability of circumpapillary RNFL measurement between localized and diffuse RNFL defects. The best circumpapillary RNFL parameter was inferior RNFL thickness (0.914) in the localized RNFL defect group, and average RNFL thickness (0.986) in the diffuse RNFL defect group.  Table 4 presents a number of previous studies on the diagnostic ability of circumpapillary RNFL thickness in glaucoma patients [4, 14–22]. In the studies of localized RNFL defects, sectoral RNFL measurements (clock-hour or inferior quadrant) were the most useful parameters [14–17]. On the other hand, in the studies that did not classify defects as localized or diffuse type, average RNFL thickness showed a relatively improved diagnostic ability [4, 18–22]. It could be inferred that the diagnostic performance of circumpapillary RNFL measurement depends on the composition of localized and diffuse RNFL defects.

It is well known that disease severity has a significant effect on the diagnostic performance of OCT [23]. Variable diagnostic performance among several studies may be due to variation in the disease severity of the subjects. In their context, disease severity was related to functional visual field loss, and not structural RNFL loss. It is, however, more reasonable to consider that the diagnostic performance of OCT is directly affected by the severity of structural damage rather than functional visual field loss, because this instrument mainly evaluates the retinal nerve fiber structure. In addition, structural damage is generally considered to precede functional alterations in glaucoma [24, 25]. The composition of localized and diffuse RNFL defects provides valuable information on structural disease severity. When interpreting the diagnostic performance of circumpapillary RNFL measurement, it is necessary to consider the composition of localized and diffuse RNFL defects as well as the composition of mild, moderate, and severe visual field loss.

The center of mass is the position where all of the mass could be considered to be located and the location of the RNFL defect can be represented as the center of the RNFL defect. The centers of diffuse RNFL defects were located significantly closer to the optic disc center than those of localized RNFL defects. Previous studies reported that the localized RNFL defects may not be detected in conventional circumpapillary RNFL measurement [6, 26]. If localized RNFL defects expand into diffuse RNFL defects as the disease advances, it can be postulated that RNFL defects expand toward the optic disc [26]. The localized RNFL defect may be missed in circumpapillary RNFL measurement due to its outward distribution. Adjustment of the circle scan diameter may be needed to improve the detecting ability for localized RNFL defects.

The RNFL deviation map is more useful than circumpapillary RNFL measurement for detecting glaucoma with localized RNFL defects (AUC, 0.991 versus 0.914; *p* = 0.002), because it could identify a RNFL defect regardless of where it is located. Several previous studies have reported similar results, finding that the RNFL deviation map significantly improves the diagnostic sensitivity for localized RNFL defects or glaucoma detection compared with conventional circumpapillary RNFL measurement [4, 14, 16]. A broader imaging area of SD-OCT provides a greater chance to find signs of RNFL damage. On the other hand, Leung et al. [5] reported that the diagnostic performance of the RNFL deviation map parameter for detecting glaucoma was similar to that of average RNFL thickness. In their study, the proportion of localized and diffuse RNFL defects was 6.9% and 85.3%, respectively. Thus, the diffuse RNFL defects were dominant among their study subjects and this characteristic might influence the results of diagnostic performance; in our study, the diffuse RNFL defect group showed no significant difference in diagnostic performance between the RNFL deviation map and circumpapillary RNFL measurement (AUC, 1.000 versus 0.986; *p* = 0.249).

In red-free photographic studies, diffuse RNFL loss is thought to be more common than focal loss, presenting in approximately 50% of cases compared to 30% with focal loss alone (approximately 20% present with both diffuse and focal loss) [1, 2]. Similarly, in recent RNFL imaging of SD-OCT, the diffuse type represented the majority of RNFL defects (Shin et al. [26], 64.1%; Leung et al. [5], 85.3%; current study, 51.1%). However, in the early phase of RNFL loss, most RNFL damage begins with localized RNFL defects involving the inferior or superior quadrants [5]. In contrast to circumpapillary RNFL thickness, the RNFL deviation map showed no significant difference in diagnostic performance between localized and diffuse RNFL defects (*p* = 0.216). Thus, RNFL deviation map analysis showed excellent and stable performance in glaucoma detection regardless of RNFL defect type.

The RNFL deviation map detects areas in which the RNFL thickness is less than the lower 95th or 99th percentile ranges. Because the prevalence rate of glaucoma is 1.9% (2010, USA), the lower 95th or 99th percentile of RNFL measurement implies overestimation or underestimation problems in detecting RNFL defects; the yellow (<5% level) coded area tends to overestimate RNFL defects or may even contain false-positive data, whereas the red (<1% level) coded area may underestimate RNFL defects [6, 9, 26]. These issues should be considered when interpreting the RNFL deviation map. To reduce discrepancy, the RNFL deviation map needs to be compared with other structural and functional tests. In addition, the definition of diffuse RNFL defect as greater than 30 degrees was arbitrary even if it was previously used in another study [5]. Depending on the definition of the diffuse, the result might be affected.

In summary, with the assistance of the RNFL deviation map, clinicians can estimate the boundary of RNFL defects and classify localized and diffuse type using topographic quantification. The composition of localized and diffuse RNFL defects could affect the diagnostic ability of circumpapillary RNFL measurement. However, the RNFL deviation map was useful in glaucoma detection regardless of RNFL defect type.

## Figures and Tables

**Table 1:** Demographic characteristics of study subjects.

	Normal	Glaucoma	*p*	Localized RNFL defects	Diffuse RNFL defects	*p*
*N*	165	139		62	71	
Age (yrs)	56.9 ± 10.8	57.1 ± 13.6	0.849^*∗*^	55.7 ± 13.2	58.0 ± 14.0	0.340^*∗*^
Gender (male : female)	88 : 77	78 : 61	0.711^†^	33 : 29	41 : 30	0.727^†^
Intraocular pressure (mmHg)	14.9 ± 2.7	14.8 ± 3.4	0.885^*∗*^	15.2 ± 3.5	14.5 ± 3.0	0.219^*∗*^
Signal strength	8.26 ± 0.93	8.06 ± 1.04	0.073^*∗*^	8.15 ± 1.17	7.83 ± 0.90	0.233^*∗*^
Refractive error (diopters)	−0.55 ± 1.48	−0.89 ± 2.08	0.107^*∗*^	−1.19 ± 2.41	−0.71 ± 1.69	0.185^*∗*^
MD (dB)	−0.66 ± 1.31	−5.26 ± 5.12	<0.001^*∗*^	−3.06 ± 3.47	−7.56 ± 5.47	<0.001^*∗*^
(MD ≥ −6 : MD < −6)	—	91 : 48	—	49 : 13	36 : 35	0.001^†^
PSD (dB)	1.89 ± 1.01	5.14 ± 4.22	<0.001^*∗*^	2.95 ± 2.18	7.35 ± 4.55	<0.001^*∗*^
Disc area (mm^2^)	2.14 ± 0.40	2.07 ± 0.43	0.126^*∗*^	2.04 ± 0.42	2.10 ± 0.45	0.466^*∗*^
Rim area (mm^2^)	1.32 ± 0.24	0.92 ± 0.26	<0.001^*∗*^	1.05 ± 0.25	0.79 ± 0.19	<0.001^*∗*^

^*∗*^Independent *t*-test.

^†^Chi-squared test.

RNFL: retinal nerve fiber layer; MD: mean deviation; PSD: pattern standard deviation.

**Table 2:** Area under ROC curve and sensitivities at fixed specificities for RNFL deviation map and circumpapillary RNFL parameters.

	Normal eyes (*n* = 165)	Eyes with localized RNFL defects (*n* = 62)	AUCs (95% CI)	Sn/Sp (Sp ≥ 90%)	Eyes with diffuse RNFL defects (*n* = 71)	AUCs (95% CI)	Sn/Sp (Sp ≥ 90%)	*p* ^*∗*^
*RNFL defect area measurements in deviation map (mm* ^*2*^)								
Red-coded	0.03 ± 0.10	1.15 ± 0.81	0.991^†^ (0.969–0.999)	97/90	4.04 ± 2.16	1.000^†^ (0.984–1.000)	100/100	0.216

*Circumpapillary RNFL thickness parameters (μm)*								
Average	99.2 ± 6.1	88.1 ± 6.6	0.894 (0.846–0.931)	68/90	72.2 ± 9.7	0.986^†^ (0.961–0.997)	97/96	<0.001
Quadrant								
Temporal	73.7 ± 8.4	64.4 ± 10.2	0.784 (0.725–0.836)	57/90	56.1 ± 9.9	0.911 (0.867–0.944)	77/90	0.004
Superior	124.4 ± 10.8	104.2 ± 14.8	0.854 (0.801–0.897)	65/90	87.1 ± 20.5	0.941 (0.902–0.967)	85/90	0.015
Nasal	70.4 ± 8.6	65.3 ± 7.9	0.676 (0.611–0.736)	25/90	63.5 ± 9.7	0.713 (0.651–0.770)	34/91	0.504
Inferior	127.8 ± 12.1	100.6 ± 16.0	0.914^†^ (0.870–0.947)	79/92	79.7 ± 22.2	0.959 (0.925–0.980)	87/92	0.138
Clock-hour								
9	57.8 ± 7.7	53.8 ± 8.1	0.660 (0.594–0.721)	19/92	49.2 ± 9.2	0.764 (0.705–0.817)	45/92	0.060
10	85.2 ± 12.2	72.9 ± 13.8	0.768 (0.707–0.821)	42/90	61.6 ± 15.7	0.879 (0.831–0.918)	67/90	0.019
11	134.4 ± 18.3	107.5 ± 23.2	0.817 (0.761–0.865)	53/90	84.8 ± 29.6	0.916 (0.873–0.948)	79/90	0.016
12	125.9 ± 20.2	106.5 ± 24.8	0.720 (0.657–0.778)	35/90	91.1 ± 26.0	0.847 (0.794–0.890)	62/90	0.012
1	112.8 ± 18.8	98.5 ± 20.3	0.708 (0.644–0.766)	34/92	85.3 ± 20.2	0.836 (0.783–0.881)	62/90	0.012
2	85.1 ± 13.7	79.1 ± 12.9	0.617 (0.550–0.681)	21/90	73.9 ± 15.0	0.729 (0.668–0.785)	42/90	0.045
3	61.3 ± 9.7	57.2 ± 9.5	0.629 (0.563–0.692)	23/90	58.7 ± 10.0	0.567 (0.501–0.631)	24/90	0.296
4	64.9 ± 10.5	59.5 ± 9.4	0.653 (0.588–0.715)	19/90	58.0 ± 10.5	0.684 (0.620–0.742)	28/90	0.582
5	99.2 ± 14.8	86.6 ± 14.8	0.730 (0.667–0.786)	39/90	74.1 ± 17.2	0.880 (0.831–0.918)	69/91	0.002
6	136.3 ± 20.9	106.1 ± 22.9	0.844 (0.790–0.889)	60/90	83.4 ± 31.8	0.905 (0.860–0.939)	80/100	0.138
7	147.9 ± 17.2	109.4 ± 27.0	0.880 (0.830–0.919)	68/95	81.7 ± 31.0	0.959 (0.925–0.981)	87/93	0.011
8	78.0 ± 12.0	66.5 ± 16.0	0.737 (0.675–0.793)	47/90	57.4 ± 14.5	0.865 (0.814–0.906)	63/91	0.012

*p* ^*∗*^			0.002^†^			0.249^†^		

^*∗*^Comparison of AUCs by the method of DeLong et al.

^†^Comparison of highest AUCs between circumpapillary RNFL thickness and RNFL defect area measurements in deviation map.

ROC: receiver operating characteristic; RNFL: retinal nerve fiber layer; AUC: area under receiver operating characteristic curve; CI: confidence interval; Sn: sensitivity; Sp: specificity.

**Table 3:** Comparison of the centers of RNFL defects between localized and diffuse RNFL defects.

	Localized RNFL defects (*n*)	Diffuse RNFL defects (*n*)	*p*
*Coordinates of RNFL defect center*			
Distance^*∗*^ (mm)	2.14 ± 0.42 (62)	1.87 ± 0.24 (71)	<0.001
Location^†^ (°)			
Superior hemifield	54.6 ± 21.7 (21)	56.3 ± 14.6 (27)	0.746
Inferior hemifield	295.3 ± 18.3 (41)	290.2 ± 15.0 (44)	0.272

^*∗*^Distance from the optic disc center to the center of RNFL defect.

^†^Angular location was measured in a clockwise direction in right eyes and in a counterclockwise direction in left eyes, with the temporal equator set at 0°.

RNFL: retinal nerve fiber layer.

**Table 4:** Previous studies on the diagnostic ability of circumpapillary RNFL thickness for localized and noncategorized RNFL defects.

Author	Year	Machine	*N*	3 best RNFL parameters^*∗*^		
				1st	2nd	3rd
*Localized RNFL defects*						
Shin et al. [14]	2013	3D-OCT 2000	64	Inferior quadrant	7 clock-hour	Average
Kim et al. [15]	2013	Cirrus HD-OCT	48	7 clock-hour	Average	Inferior quadrant
		3D-OCT 2000	48	7 clock-hour	Inferior quadrant	Average
Kim et al. [16]	2010	Cirrus HD-OCT	66	Inferior quadrant	Average	Superior quadrant
		Stratus OCT	66	Inferior quadrant	Average	Superior quadrant
Jeoung and Park [17]	2010	Cirrus HD-OCT	55	Inferior quadrant	7 clock-hour	11 clock-hour
		Stratus OCT	55	7 clock-hour	11 clock-hour	Inferior quadrant
*Noncategorized RNFL defects*						
Leung et al. [4]	2010	Cirrus HD-OCT	121	Inferior quadrant	Average	7 clock-hour
		Stratus OCT	121	7 clock-hour	Average	Inferior quadrant
Park et al. [18]	2009	Cirrus HD-OCT	100	Inferior quadrant	Average	7 clock-hour
		Stratus OCT	100	Inferior quadrant	Average	7 clock-hour
Leung et al. [19]	2009	Cirrus HD-OCT	83	Superior quadrant	Average	Inferior quadrant
		Stratus OCT	83	Average	Superior quadrant	7 clock-hour
Lu et al. [20]	2008	Stratus OCT	89	Average	Inferior quadrant	Superior quadrant
Medeiros et al. [21]	2005	Stratus OCT	88	Average	Inferior quadrant	7 clock-hour
Huang and Chen [22]	2005	Stratus OCT	89	Inferior quadrant	Average	7 clock-hour

^*∗*^Diagnostic ability was evaluated by area under receiver operating characteristic curve.

RNFL: retinal nerve fiber layer.
